# MTMF-Grid: A multi-task multi-modal fusion model for operational forecasting and decision support in power grids

**DOI:** 10.1371/journal.pone.0343511

**Published:** 2026-03-12

**Authors:** Dongyu Zhang, Biao Shen, Peng Li, Pengcheng Wang, Yang Sheng, Yuqi Bing

**Affiliations:** 1 State Grid Jiangsu Electric Power Co., Ltd., Nanjing China; 2 Jiangsu Xinshun Energy Development Co., Ltd., Nanjing, China; 3 Wuxi Guangying Group Co., Ltd., Wuxi, China; 4 State Grid Nanjing Power Supply Company, Nanjing, China; Dr Shakuntala Misra National Rehabilitation University, INDIA

## Abstract

Power grid strategic emerging business investment features multi-objective coupling and multi-source heterogeneous data. It requires simultaneous completion of regression and classification tasks, making traditional single-task or single-modal assessment methods inadequate for precise decision-making. This study proposes a multi-task multi-modal fusion model (MTMF-Grid) for operational forecasting and decision support in strategic emerging power grid investments. MTMF-Grid leverages operational data proxies to support investment decision-making, rather than directly predicting financial returns. MTMF-Grid adopts a modular architecture with three core mechanisms: a task-adaptive Transformer to balance general and task-specific feature expression, a Cross-Fusion Gating Mechanism (CFGM) for dynamic multi-modal fusion and robustness to modal missing scenarios, and a loss variance-based mechanism to dynamically adjust task weights and alleviate gradient conflicts. Experiments on SEWA (Sharjah Electricity and Water Authority dataset) and OPSD (Open Power System Data) datasets show MTMF-Grid outperforms mainstream baseline models. It achieves 3.06% MAPE (Mean Absolute Percentage Error) for hourly electricity price prediction, 0.915 accuracy for load fluctuation risk classification. This study presents a comprehensive framework for supporting strategic decision-making in power grid investment through operational forecasting and multi-modal data integration.

## Introduction

Amid the construction of new power systems, power grid investment is shifting toward strategic emerging projects such as new energy supporting power grids, energy storage stations, and microgrids [1,2]. These projects exhibit characteristics of multi-objective coupling and multi-source heterogeneous data, requiring simultaneous completion of regression tasks such as investment return rate prediction and classification tasks such as technical feasibility evaluation [3]. Traditional single-task or single-modal evaluation methods can no longer meet the needs of precise decision-making. Constructing an intelligent evaluation model that balances multi-task collaboration and multi-modal fusion has become a core technical requirement for scientific power grid investment decision-making.

The development of deep learning technology has provided a new path for complex evaluation scenarios, and relevant research has made remarkable progress in both power grid evaluation and multi-task multi-modal learning fields. In the field of power grid evaluation, time-series models such as LSTM and GRU are used to capture long-range dependencies of power demand and investment returns [4,5], CNN architectures realize technical feature extraction and feasibility classification [6], and Transformer models strengthen multi-index correlation modeling through self-attention mechanisms [7]. In the field of multi-task learning, the architecture of shared bottom networks and task-specific output heads is widely used in engineering evaluation, improving generalization ability through information sharing among tasks [8,9]; in terms of multi-modal fusion, attention mechanisms and gating strategies have become mainstream, realizing feature integration of heterogeneous data such as financial, environmental, and technical data [10,11]. For example, some studies have constructed multi-task models to jointly realize power grid project risk assessment and return prediction, fusing multi-source data through feature concatenation [12,13]; other studies have adopted static attention weight allocation mechanisms to improve fusion efficiency [14]. These studies have verified the feasibility of the multi-task multi-modal approach, but there are still key technical shortcomings in meeting the complex needs of strategic emerging power grid investment projects. The core deficiencies of existing research focus on three aspects: first, the lack of adaptability in multi-task feature modeling, where the unified feature extraction architecture cannot meet the specific needs of mixed regression and classification tasks, resulting in insufficient pertinence of feature expression [15]; second, the lack of flexibility in multi-modal fusion mechanisms, where static weights or simple concatenation are difficult to capture dynamic correlations among modalities, and the robustness to modal missing scenarios is insufficient [16]; third, the imperfection of training optimization strategies, where the traditional fixed-weight loss function is prone to gradient conflicts among tasks, leading to training imbalance. These problems severely limit the in-depth application of deep learning technology in the evaluation of emerging power grid investment projects, and there is an urgent need to construct a more scenario-adaptive technical framework.

To solve the above problems, this paper proposes a multi-task multi-modal fusion model MTMF-Grid, specifically for the comprehensive evaluation of strategic emerging power grid investment projects. Based on the modular design idea of deep learning, the model constructs a three-level core architecture, including task-adaptive feature modeling, cross-modal dynamic fusion, and adaptive training optimization. It adapts to the needs of different evaluation tasks through task-specific self-attention mechanisms, realizes dynamic integration of heterogeneous data using cross-modal gating fusion mechanisms, and introduces adaptive weight adjustment mechanisms to balance the training rhythm of multi-task. The three work synergistically to achieve in-depth mining of multi-source data and efficient collaborative learning of multi-objective tasks. Fully combining the industry characteristics of power grid project evaluation, the model optimizes the network structure and training strategy, and can accurately output multi-dimensional evaluation results such as investment return rate, technical feasibility, and environmental impact level, providing comprehensive technical support for investment decision-making.

The core contributions of this paper are as follows:

- A task-adaptive Transformer feature modeling module is proposed. Through task-specific self-attention mechanisms and multi-head attention structures, it adaptively captures the differences in feature needs of different evaluation tasks, solves the imbalance between the generality and task specificity of feature expression in traditional models, and improves the adaptability of deep learning models to multi-task scenarios.
- A Cross-Fusion Gating Mechanism (CFGM) is designed. Integrating cross-modal cross-attention and gating adjustment logic, it constructs a dynamically weighted fusion architecture for multi-source heterogeneous data. Meanwhile, a modal attention mask and completion mechanism are introduced to enhance the robustness of deep learning models to modal missing scenarios.
- An adaptive task weighting mechanism based on loss variance is introduced. It dynamically balances the training losses of mixed regression and classification tasks, alleviates the gradient conflict problem in multi-task training, ensures the balanced optimization of each evaluation task, and improves the overall evaluation performance and generalization ability of deep learning models.

## Related work

### Research on power grid investment project evaluation models

In the intelligent development of power grid investment project evaluation, data-driven models have gradually replaced traditional subjective evaluation methods as the mainstream research direction. In early studies, evaluation schemes based on time-series models such as LSTM and GRU were widely applied. By mining the temporal patterns of historical power load and investment data, combined with data preprocessing techniques including sliding windows and feature differencing, these models achieved accurate prediction of economic indicators such as investment return rates, verifying their effectiveness in numerical regression tasks [17]. For classification tasks like technical feasibility evaluation, some studies adopted CNN architectures to extract local key features of project technical parameters. Through the end-to-end learning mode of deep learning, classification accuracy was significantly improved, but such models can only adapt to a single task type and cannot simultaneously handle the mixed evaluation requirements of regression and classification [18].

In recent years, Transformer models have been used to construct multi-dimensional evaluation frameworks leveraging their advantages in modeling long-range correlations among multiple indicators. These models can capture complex dependencies between indicators such as technical maturity and policy adaptability. However, most of these models adopt single-modal data input and fail to effectively integrate heterogeneous information including financial statements, environmental monitoring data, and power demand data, with insufficient robustness to small-sample data [19]. Additionally, multi-source data evaluation methods based on Deep Neural Networks (DNN) have been proposed, which improve evaluation accuracy by fusing technical, economic, and environmental data [20]. Nevertheless, these models lack adaptive design for task specificity and cannot meet the comprehensive evaluation needs of strategic emerging projects. The aforementioned studies provide data-driven technical ideas for power grid evaluation but fail to address the core issues of multi-task collaboration and deep multi-modal fusion, complementing the task-adaptive feature modeling scheme proposed in this paper.

### Application of multi-task learning in engineering evaluation

Multi-task learning achieves collaborative optimization through information sharing among tasks and has demonstrated significant advantages in the field of engineering evaluation. In existing research, multi-task architectures based on hard parameter sharing have been applied to power system-related evaluations. By sharing the underlying CNN network, these architectures simultaneously complete equipment fault diagnosis and operational risk classification tasks [21]. Utilizing the correlation between tasks, they reduce redundant parameters and improve model generalization ability and training efficiency. However, this architecture adopts a unified feature extraction logic and cannot adapt to the specific needs of mixed regression and classification tasks.

To enhance task adaptability, some studies have adopted soft parameter sharing mechanisms. On the basis of sharing a Transformer encoder, independent decoders and loss functions are set for different tasks, enabling the model to have a certain degree of flexibility in handling tasks such as return prediction and risk assessment [22]. Nevertheless, the shared features output by the encoder still lack specificity, leading to limited performance improvement for different types of tasks. In recent years, multi-task learning frameworks have further integrated advanced technologies such as attention mechanisms and generative adversarial networks. Through dynamic weight allocation and adversarial training, they strengthen the mining of inter-task correlations and feature expression capabilities [23]. However, in the scenario of power grid investment project evaluation, an adaptive feature learning mechanism for mixed tasks has not yet been formed, and the temporal nature and strong coupling of power industry data have not been fully considered.In contrast, the task-adaptive Transformer module proposed in this paper dynamically adjusts feature extraction strategies through task-specific self-attention mechanisms, effectively solving the balance between feature generality and specificity in mixed task scenarios and filling the gap in scenario adaptability of existing research.

### Research on multi-modal fusion technology

Multi-modal fusion technology provides an effective path for integrating multi-source heterogeneous data in power grid evaluation. Existing methods can be divided into three categories: data-level fusion, feature-level fusion, and decision-level fusion. In terms of data-level fusion, some studies adopt feature concatenation to integrate financial data and technical parameters [24,25]. After eliminating dimensional differences through standardization and normalization, the fused features are input into MLP models for investment evaluation. However, this method fails to consider dynamic correlations between modalities, is susceptible to interference from redundant information, and sensitive to noisy data, resulting in limited fusion effects.

Feature-level fusion often uses static attention mechanisms, which assign fixed weights to different types of data like finance, environment, and technology. This helps improve evaluation accuracy by emphasizing important information. But these fixed weights can’t adjust when data changes dynamically and don’t perform well in complex situations like technology updates or policy changes [26]. In the power industry, some approaches try to fix this by adding cross-modal adaptation layers, altering basic architectures. They use methods like linear transformation and attention mapping to bring different data sources, such as text and images, into a common space, improving multi-modal analysis [27,28]. One study also showed how multi-modal fusion can work in power systems by combining data like voiceprints, vibrations, and infrared signals to create digital twins of equipment [29,30]. However, these methods have some key problems. They don’t capture dynamic connections well, struggle with missing data, and have high computational costs. Also, there is no tailored solution for evaluating power grid investment projects. The core issues with existing methods are their inability to adapt to dynamic inter-modal relationships, inefficiencies in handling missing data, and the lack of robust solutions for the specific needs of power grid investment evaluation.

The Cross-Fusion Gating Mechanism (CFGM) proposed here addresses these issues by dynamically allocating weights and using attention mask strategies to fill in missing data. It improves adaptability to missing modalities and fusion efficiency, offering better flexibility for different scenarios compared to static fusion or simple concatenation methods.

## Methodology

### Overall model architecture

**Fig 1:**
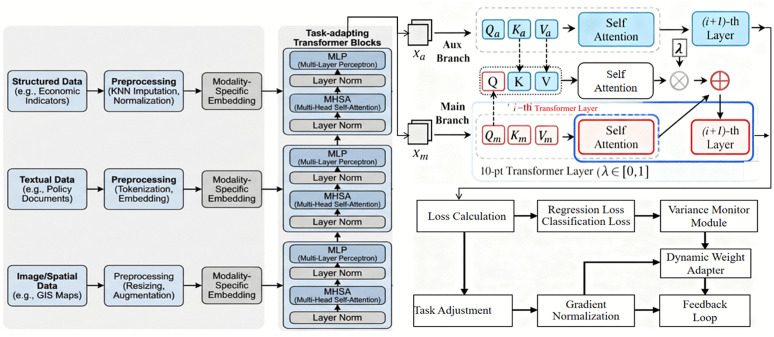
Overall architecture of the MTMF-Grid model for power grid strategic emerging investment project evaluation. The framework sequentially integrates: (1) A multi-source data preprocessing pipeline; (2) Task-adapting Transformer blocks for feature extraction; (3) A dual-branch attention module (with self-attention and layer stacking) to refine task-specific features; and (4) A loss variance-based adaptive training optimization mechanism to balance the training of mixed regression and classification tasks.

The MTMF-Grid model integrates multiple components to handle both regression and classification tasks in evaluating power grid investment projects. It includes a feature modeling layer that uses a Transformer to adaptively capture task-specific features. The model employs a Cross-Fusion Gating Mechanism to dynamically fuse multi-modal data, ensuring robust performance even when some modalities are missing. Separate output heads are designed for regression and classification tasks, allowing the model to generate accurate predictions for both types of tasks. The training optimization layer adjusts task weights based on loss variance, ensuring balanced optimization during multi-task learning. Figure 1 illustrates the overall architecture of the MTMF-Grid model.

### Task-adaptive transformer feature modeling module

The core goal of the task-adaptive Transformer feature modeling module is to address the problem that traditional unified feature extraction architectures cannot adapt to mixed regression and classification tasks. By designing dedicated feature learning channels for different types of tasks, it achieves accurate separation and efficient fusion of shared features and task-specific features, ultimately outputting high-quality features with both generality and pertinence. The input of this module is the preprocessed multi-modal data feature matrix $𝐗\in {\mathbb{R}}^{N\times D}$ from the input layer, where N represents the number of samples (e.g., different power grid investment projects) and D represents the original feature dimension of each sample (combining dimensions of multi-modal indicators such as finance, technology, environment, and power demand). The input features have undergone preprocessing operations such as standardization and preliminary missing value imputation to ensure uniform data format and reliable quality.

To enable the model to capture temporal dependencies in the data (e.g., temporal variation patterns of power demand and long-term trends of investment returns) and map original features to a high-dimensional feature space that the model can process efficiently, the input features are first subjected to a fusion operation of linear projection and positional encoding.


$${𝐗}_{\text{proj}}=𝐗\cdot {𝐖}_{\text{proj}}+𝐏$$ (1)

Here, ${𝐖}_{\text{proj}}\in {\mathbb{R}}^{D\times D_{\text{model}}}$ is a learnable linear projection weight matrix, which maps the original D-dimensional features to the $D_{\text{model}}$-dimensional hidden layer feature space of the model; $𝐏\in {\mathbb{R}}^{N\times D_{\text{model}}}$ is a positional encoding matrix generated using sine-cosine positional encoding. By injecting positional information, the model can perceive the temporal order of samples or features (for non-temporal features, positional encoding is used to distinguish the relative positions of different indicators). $D_{\text{model}}$ is the model’s hidden layer dimension, set to 512 based on experimental validation. The enhanced feature matrix ${𝐗}_{\text{proj}}\in {\mathbb{R}}^{N\times D_{\text{model}}}$ output by this step not only retains the core information of the original multi-modal data but also possesses temporal perception capability, laying a foundation for subsequent task-adaptive feature learning.

For the two core tasks in power grid investment evaluation—regression tasks (e.g., investment return rate prediction, operating cost estimation) and classification tasks (e.g., technical feasibility grade evaluation, environmental impact grade classification)—the module designs an independent self-attention mechanism for each task t (where t=1 denotes regression tasks and t=2 denotes classification tasks), namely task-specific self-attention. Its core is to enable the model to targetedly learn the key feature correlations required by the task through dedicated projection weight matrices. First, the query matrix (Query), key matrix (Key), and value matrix (Value) for task t are calculated, all of which are transformed from the enhanced feature matrix ${𝐗}_{\text{proj}}$ using different task-specific projection weight matrices to ensure that the feature mapping direction of each task matches its own needs.


$${𝐐}_{t}={𝐗}_{\text{proj}}\cdot {𝐖}_{Q}^{t}$$ (2)

$${𝐊}_{t}={𝐗}_{\text{proj}}\cdot {𝐖}_{K}^{t}$$ (3)

$${𝐕}_{t}={𝐗}_{\text{proj}}\cdot {𝐖}_{V}^{t}$$ (4)

Here, ${𝐖}_{Q}^{t},{𝐖}_{K}^{t},{𝐖}_{V}^{t}\in {\mathbb{R}}^{D_{\text{model}}\times D_{k}}$ are all learnable task-specific projection weight matrices for task t. $D_{k}$ is the dimension of the attention head, set to $D_{\text{model}}/h$ (where h is the number of attention heads) to ensure an appropriate dimension for each attention head, improving computational efficiency and feature expression capability. Based on the query matrix and key matrix, the attention score for task t is calculated to measure the correlation strength between different features, i.e., the importance contribution of one feature to another.


$${𝐀}_{t}=\text{Softmax}\left(\frac{{𝐐}_{t}\cdot {𝐊}_{t}^{⊤}}{\sqrt{D_{k}}}\right)$$ (5)

The linear projection with positional encoding, task-specific Q/K/V projection, and scaled dot-product attention calculation are built on the classic Transformer architecture and its multi-task learning variants, where the sine-cosine positional encoding and scaled attention mechanism ensure effective capture of temporal dependencies in power grid data [31–33].

In the formula, ${𝐐}_{t}\cdot {𝐊}_{t}^{⊤}$ calculates the similarity matrix between queries and keys. Dividing by $\sqrt{D_{k}}$ scales the values to avoid excessively large similarity values due to a large $D_{k}$, which could affect the gradient stability of the Softmax function. The Softmax function normalizes along the feature dimension so that the sum of attention scores is 1. The resulting attention matrix ${𝐀}_{t}\in {\mathbb{R}}^{N\times N}$ has each element $A_{t}(i,j)$ representing the attention weight of the j-th sample (or feature) to the i-th sample (or feature). Multiplying the attention matrix by the value matrix yields the single-head self-attention output for task t, realizing weighted feature fusion based on feature correlation strength.


$${𝐕}_{t}^{\text{attn}}={𝐀}_{t}\cdot {𝐕}_{t}$$ (6)

This output ${𝐕}_{t}^{\text{attn}}\in {\mathbb{R}}^{N\times D_{k}}$ has initially captured the feature correlation information required by task t, but a single attention head has a limited perspective and cannot fully explore complex feature dependencies.


$${𝐕}_{t}^{\text{multi}}=\text{Concat}\left({𝐕}_{t,1}^{\text{attn}},{𝐕}_{t,2}^{\text{attn}},\ldots ,{𝐕}_{t,h}^{\text{attn}}\right)\cdot {𝐖}_{O}^{t}$$ (7)

In this equation, h represents the number of attention heads, which is set to 8 based on experiments. Each attention head has its own weight matrices ${𝐖}_{Q}^{t},{𝐖}_{K}^{t},{𝐖}_{V}^{t}$, which allow the model to capture feature correlations from different angles. ${𝐕}_{t,i}^{\text{attn}}$ (for i=1,2,...,h) is the output from the i-th attention head. The operation Concat(·) concatenates the outputs from all 8 heads along the feature dimension, resulting in a feature matrix of size $N\times (h\cdot D_{k})$. The matrix ${𝐖}_{O}^{t}\in {\mathbb{R}}^{h\cdot D_{k}\times D_{\text{model}}}$ projects the concatenated features back to $D_{\text{model}}$ dimensions, so that the dimensions match for the next calculations. This multi-head attention helps the model capture both local feature correlations and global dependencies, improving the model’s ability to distinguish task-specific features.

To further improve the model’s ability to express nonlinear features and solve the gradient vanishing problem in deep networks, a Feed-Forward Network (FFN) with a residual connection is introduced. This adds nonlinear transformation and gradient optimization to the output of the multi-head attention.


$${𝐅}_{t}=\text{FFN}\left({𝐕}_{t}^{\text{multi}}+{𝐗}_{\text{proj}}\right)$$ (8)

$$\text{FFN}(\cdot )=\max(0,𝐙\cdot {𝐖}_{1}+{𝐛}_{1})\cdot {𝐖}_{2}+{𝐛}_{2}$$ (9)

The multi-head attention concatenation strategy, residual connection, and two-layer ReLU-based FFN structure follow the optimized Transformer block design, which has been widely validated in power system time-series analysis tasks for alleviating gradient vanishing and enhancing nonlinear feature expression [23,34,35].

In the formula, $𝐙={𝐕}_{t}^{\text{multi}}+{𝐗}_{\text{proj}}$ is the feature matrix after residual connection; ${𝐖}_{1}\in {\mathbb{R}}^{D_{\text{model}}\times 4D_{\text{model}}}$ and ${𝐖}_{2}\in {\mathbb{R}}^{4D_{\text{model}}\times D_{\text{model}}}$ are learnable weight matrices of the feed-forward neural network. The first layer expands the feature dimension from $D_{\text{model}}$ to $4D_{\text{model}}$, and the second layer compresses it back to $D_{\text{model}}$, enhancing nonlinear expression capability through dimension expansion; ${𝐛}_{1}\in {\mathbb{R}}^{4D_{\text{model}}}$ and ${𝐛}_{2}\in {\mathbb{R}}^{D_{\text{model}}}$ are bias vectors; max(0,·) is the ReLU activation function, introducing nonlinear transformation to enable the model to learn complex feature mapping relationships.

Finally, the module outputs the task-specific feature vector ${𝐅}_{t}\in {\mathbb{R}}^{N\times D_{\text{model}}}$ for task t. For regression tasks, this feature vector emphasizes features related to numerical prediction (e.g., investment scale, load trends); for classification tasks, it strengthens features related to category determination. Meanwhile, since all tasks share the initial linear projection and positional encoding steps, ${𝐅}_{t}$ also contains shared features common to multiple tasks, achieving the organic combination of shared and specific features. This not only ensures information sharing among multiple tasks but also meets the specific needs of different tasks, providing high-quality and highly adaptive feature inputs for the subsequent cross-modal fusion layer.

### Cross-Modal Fusion Gating Mechanism (CFGM)

The core of the Cross-Modal Fusion Gating Mechanism (CFGM) is to process multi-modal features output by the task-adaptive Transformer module, which specifically include financial feature ${𝐅}_{f}$, grid operation (technical) feature ${𝐅}_{o}$, environmental feature ${𝐅}_{e}$, and power demand feature ${𝐅}_{d}$ (all with dimensions ${\mathbb{R}}^{N\times D_{\text{model}}}$). These features are derived from SEWA and OPSD datasets: ${𝐅}_{d}$ is extracted from SEWA’s hourly load, load peak, and load volatility; ${𝐅}_{o}$ is derived from OPSD’s transmission capacity, load rate, and reserve capacity; ${𝐅}_{f}$ is based on OPSD’s electricity price; ${𝐅}_{e}$ is constructed from OPSD’s renewable energy generation ratio. These features are used as proxies for investment evaluation metrics, such as annual investment returns, based on operational data.

First, CFGM sets a main branch and an auxiliary branch for each target modality *m* (where m∈{f,o,e,d} corresponds to financial, grid operation, environmental, and power demand modalities respectively): the main branch is responsible for mining intra-modal feature dependencies, performing self-attention calculation on the target modality ${𝐅}_{m}$ to extract key correlation information within the modality (e.g., temporal correlation of electricity prices in financial modality, correlation between load rate and reserve capacity in grid operation modality). The calculation process is as follows:


$${𝐐}_{m}^{\text{self}}={𝐅}_{m}\cdot {𝐖}_{Q}^{m}$$ (10)

$${𝐊}_{m}^{\text{self}}={𝐅}_{m}\cdot {𝐖}_{K}^{m}$$ (11)

$${𝐕}_{m}^{\text{self}}={𝐅}_{m}\cdot {𝐖}_{V}^{m}$$ (12)

Here, ${𝐖}_{Q}^{m}$, ${𝐖}_{K}^{m}$, and ${𝐖}_{V}^{m}$ are learnable projection weights exclusive to the target modality *m*, converting ${𝐅}_{m}$ into query, key, and value matrices required for self-attention. In the power demand modality ${𝐅}_{d}$, the projection weights are optimized to emphasize the temporal correlation of load data; in the grid operation modality ${𝐅}_{o}$, they focus on the inherent association between grid operation parameters. Based on these matrices, the intra-modal attention score and output are calculated:


$${𝐀}_{m}^{\text{self}}=\text{Softmax}\left(\frac{{𝐐}_{m}^{\text{self}}\cdot ({𝐊}_{m}^{\text{self}}{)}^{⊤}}{\sqrt{D_{k}}}\right)$$ (13)

$${𝐎}_{m}^{\text{self}}={𝐀}_{m}^{\text{self}}\cdot {𝐕}_{m}^{\text{self}}$$ (14)

Among them, $D_{k}=D_{\text{model}}/h$ (where h=8 is the number of attention heads, consistent with the task-adaptive Transformer module) is used to scale the attention score to avoid numerical overflow, and the Softmax function normalizes the attention score in the sample dimension. The finally obtained ${𝐎}_{m}^{\text{self}}$ is an enhanced intra-modal feature that retains the core information of the modality itself (e.g., key fluctuation patterns of power demand, critical threshold values of grid operation parameters).

The auxiliary branch focuses on inter-modal correlations, taking the target modality *m* as the center and introducing all other modalities *n* (n∈{f,o,e,d},n≠m) as auxiliary inputs to capture the dependency between the target modality and auxiliary modalities through cross-attention—this is particularly important for power grid investment evaluation (e.g., the correlation between power demand and electricity price, the association between renewable energy generation ratio and grid load rate). For a single auxiliary modality *n*, the cross-attention calculation is:


$${𝐐}_{m}^{\text{cross}}={𝐅}_{m}\cdot {𝐖}_{Q}^{m}$$ (15)

$${𝐊}_{n}^{\text{cross}}={𝐅}_{n}\cdot {𝐖}_{K}^{n}$$ (16)

$${𝐕}_{n}^{\text{cross}}={𝐅}_{n}\cdot {𝐖}_{V}^{n}$$ (17)

Here, ${𝐖}_{K}^{n}$ and ${𝐖}_{V}^{n}$ are projection weights of the auxiliary modality *n*, which are optimized to highlight the features of *n* that are relevant to the target modality *m* (e.g., when *m* is the power demand modality, the projection weights of the financial modality n=f emphasize electricity price features related to load changes). The query matrix reuses ${𝐐}_{m}^{\text{cross}}$ from the main branch to maintain feature alignment. Based on this, the cross-attention output is calculated:


$${𝐀}_{m,n}^{\text{cross}}=\text{Softmax}\left(\frac{{𝐐}_{m}^{\text{cross}}\cdot ({𝐊}_{n}^{\text{cross}}{)}^{⊤}}{\sqrt{D_{k}}}\right)$$ (18)

$${𝐎}_{m,n}^{\text{cross}}={𝐀}_{m,n}^{\text{cross}}\cdot {𝐕}_{n}^{\text{cross}}$$ (19)

Since the target modality *m* corresponds to 3 auxiliary modalities (excluding itself), it is necessary to average the cross-attention outputs of all auxiliary modalities to obtain the comprehensive inter-modal correlation feature of the target modality *m*:


$${𝐎}_{m}^{\text{cross}}=\frac{1}{3}\sum_{n\neq m}{𝐎}_{m,n}^{\text{cross}}$$ (20)

This averaging operation integrates the complementary information from multiple auxiliary modalities, ensuring that the inter-modal features are more robust. Next, CFGM realizes dynamic fusion of dual-branch features through a gating unit and adapts to modal missing scenarios. If the target modality *m* is complete, mark ${𝐒}_{m}=1$; if the modality is missing, mark ${𝐒}_{m}=0$. Then, calculate the gating weight vector:


$${𝐆}_{m}={𝐒}_{m}\cdot \text{Sigmoid}\left(\text{MeanPooling}({𝐎}_{m}^{\text{self}})\cdot {𝐖}_{g}+{𝐛}_{g}\right)$$ (21)

Here, ${𝐒}_{m}$ is a binary indicator for modality completeness: it is 1 if the modality is complete and 0 if it is missing. ${𝐆}_{m}$ represents the gating weight vector, which is used to dynamically adjust the importance of the modality. When the modality is complete, ${𝐆}_{m}$ is close to 1, prioritizing the main branch; when missing, ${𝐆}_{m}$ is close to 0, relying on the auxiliary branch to compensate for the missing data.

Based on the gating weight, fuse the main and auxiliary branch features and add a residual connection to obtain the branch fused feature of a single modality, ensuring that the original modal information is not lost:


$${𝐅}_{m}^{\text{branch}}={𝐆}_{m}⊙{𝐎}_{m}^{\text{self}}+(1-{𝐆}_{m})⊙{𝐎}_{m}^{\text{cross}}+{𝐅}_{m}$$ (22)

Here, ⊙ is the element-wise product, and the residual connection ${𝐅}_{m}$ retains the original feature information of the modality, avoiding information loss during the fusion process while improving the stability of gradient propagation—this is crucial for maintaining the physical meaning of power grid-related features. To prevent noise or redundancy in cases of missing modalities, we explicitly mask the features of absent modalities to zero. This ensures that the residual connection does not introduce any unwanted information into the fused representation when a modality is missing. By forcing missing modality features to zero, we guarantee that the residual connection only contributes valid data from available modalities, thus preventing feature degradation in incomplete data scenarios.

Finally, concatenate the branch fused features of all four modalities by dimension, and then map the high-dimensional features back to the $D_{\text{model}}$ dimension through linear projection to obtain the global fused feature that integrates multi-modal information:


$${𝐅}_{\text{fusion}}=\text{Concat}({𝐅}_{f}^{\text{branch}},{𝐅}_{o}^{\text{branch}},{𝐅}_{e}^{\text{branch}},{𝐅}_{d}^{\text{branch}})\cdot {𝐖}_{\text{fusion}}+{𝐛}_{\text{fusion}}$$ (23)

The intra-modal self-attention, inter-modal cross-attention and weighted fusion strategies are designed based on the multi-modal attention fusion framework, and the gating mechanism combined with missing modality masking refers to the adaptive fusion method for heterogeneous power grid data [10,27,30].

Among them, ${𝐖}_{\text{fusion}}\in {\mathbb{R}}^{4D_{\text{model}}\times D_{\text{model}}}$ and ${𝐛}_{\text{fusion}}\in {\mathbb{R}}^{N\times D_{\text{model}}}$ are learnable parameters of the projection layer, which map the concatenated $4D_{\text{model}}$-dimensional features to the unified $D_{\text{model}}$-dimensional space. The output ${𝐅}_{\text{fusion}}$ not only integrates the core information of financial, grid operation, environmental, and power demand modalities but also maintains effective output when some modalities are missing. It provides comprehensive and robust feature support for subsequent power grid investment evaluation tasks such as annual investment income prediction and technical feasibility grade classification, ensuring that the model can make accurate and reliable decisions based on multi-source information.

### Task output layer design

The core of the task output layer is to map the global fused feature ${𝐅}_{\text{fusion}}\in {\mathbb{R}}^{N\times D_{\text{model}}}$ output by the cross-modal fusion gating mechanism to the final results of two core tasks in power grid investment evaluation (regression and classification). It designs dedicated output heads for the output characteristics of different tasks to ensure the accuracy of results and consistency of physical meaning, fully aligning with the actual needs of investment decision-making.


$${\hat{y}}_{r}={𝐅}_{\text{fusion}}^{\text{bn}}\cdot {𝐖}_{r}+{𝐛}_{r}$$ (24)

Here, ${𝐖}_{r}\in {\mathbb{R}}^{D_{\text{model}}\times 1}$ and ${𝐛}_{r}\in {\mathbb{R}}^{N}$ are learnable parameters that help fit the linear relationship between the multi-modal fused features (like electricity price, load volume, transmission capacity) and the operational metrics. The operational data (such as electricity price and load) serve as proxies for the financial returns in investment evaluation. These metrics support investment decision-making, but do not directly represent IRR or NPV. To improve the accuracy and speed up training, batch normalization is applied to the fused features before they go into the fully connected layer:


$${𝐅}_{\text{fusion}}^{\text{bn}}=\text{BatchNorm}({𝐅}_{\text{fusion}})$$ (25)

This normalization helps reduce the effect of feature distribution changes (like differences in electricity prices across regions), making sure the model trains consistently across different datasets. The final output ${\hat{y}}_{r}\in {\mathbb{R}}^{N}$ is the predicted annual income for each sample (e.g., a regional grid expansion project), giving a direct numerical assessment for investment decisions.

For classification tasks in power grid investment, like evaluating technical feasibility grades (high, medium, low feasibility, or labels 1, 2, 3), the output head uses a fully connected layer and Softmax activation to turn the fused features into a probability distribution over categories. First, the fully connected layer turns the $D_{\text{model}}$-dimensional fused features into a C-dimensional category score vector (with C=3 for the three feasibility grades):


$${𝐳}_{c}={𝐅}_{\text{fusion}}\cdot {𝐖}_{c}+{𝐛}_{c}$$ (26)

Here, ${𝐖}_{c}\in {\mathbb{R}}^{D_{\text{model}}\times C}$ and ${𝐛}_{c}\in {\mathbb{R}}^{N\times C}$ are learnable parameters. They are optimized to highlight the important features for grading feasibility, such as grid load rate, reserve capacity, and load fluctuations. The Softmax function then turns the score vector into a probability distribution, showing how confident the model is in each classification result:


$${\hat{y}}_{c}=\text{Softmax}({𝐳}_{c})=\frac{\exp({𝐳}_{c})}{\sum_{k=1}^{C}\exp({𝐳}_{c,k})}$$ (27)

The fully connected layer-based regression output and classification output with Softmax activation follow the standard task-specific head design for multi-task learning, and the batch normalization operation refers to the optimization strategy for power grid data regression and classification tasks [12,35].

Here, ${\hat{y}}_{c}\in {\mathbb{R}}^{N\times C}$, and each element ${\hat{y}}_{c,i,k}$ represents the probability that the i-th project belongs to the k-th feasibility grade. The sum of the probabilities for all categories equals 1. For example, if a project has ${\hat{y}}_{c,i,1}=0.85$, it means there’s an 85% chance it’s a high-feasibility project, helping prioritize investments.

This design enables the model to provide direct and reliable judgment basis for the comprehensive evaluation of power grid investment projects, bridging the gap between feature learning and practical investment decision-making.

### Adaptive training optimization mechanism based on loss variance

The core goal of the adaptive training optimization mechanism based on loss variance is to solve the problems of gradient conflict and unbalanced training rhythm between regression (annual investment income prediction) and classification (technical feasibility grade evaluation) tasks in power grid investment multi-task training. By dynamically adjusting the loss weights of each task, it balances the training process of different tasks and ensures that the model can achieve optimal performance on both types of tasks, avoiding the situation where one task dominates training due to differences in loss scales (e.g., income prediction loss with large numerical values overwhelming classification loss). This mechanism takes the single-task loss of the task output layer as input, perceives changes in training status through loss variance, and constructs a dynamically weighted joint loss function to realize the collaborative optimization of model parameters.

First, loss functions are defined for the two core tasks respectively, tailored to the characteristics of power grid investment evaluation: For the regression task, the mean squared error (MSE) loss function is adopted to measure the deviation between the predicted income and the true income. The calculation is as follows:


$${ℒ}_{r}=\frac{1}{N}\sum_{i=1}^{N}({\hat{y}}_{r,i}-y_{r,i}{)}^{2}$$ (28)

where ${\hat{y}}_{r,i}$ is the predicted annual income of the *i*-th sample, $y_{r,i}$ is the corresponding true income, and *N* is the number of samples. This loss function effectively penalizes large prediction deviations (e.g., overestimating income by millions of EUR), promoting the convergence of regression results to the true values and ensuring the reliability of financial evaluation.

The cross-entropy loss function is adopted to optimize the discrimination accuracy of discrete feasibility grades. The calculation is as follows:


$${ℒ}_{c}=-\frac{1}{N}\sum_{i=1}^{N}\sum_{k=1}^{C}y_{c,i,k}\log({\hat{y}}_{c,i,k})$$ (29)

where C=3 is the number of feasibility grades, $y_{c,i,k}$ is the one-hot encoded true label of the *i*-th sample in the *k*-th grade (e.g., $y_{c,i,1}=1$ indicates a high-feasibility project), and ${\hat{y}}_{c,i,k}$ is the corresponding predicted probability. By maximizing the logarithmic probability of the true grade, this loss function enhances the model’s ability to distinguish between different feasibility levels, especially for boundary cases (e.g., projects with load rates around 80).

To real-time capture the training stability of each task (e.g., whether income prediction loss fluctuates sharply due to regional electricity price differences), a sliding window is introduced to calculate the variance of task loss, quantifying the fluctuation degree of the loss:


$$\sigma_{t}^{2}=\frac{1}{T}\sum_{s=t-T+1}^{t}({ℒ}_{s}-{\bar{ℒ}}_{t}{)}^{2}$$ (30)

where $\sigma_{t}^{2}$ is the task loss variance at the *t*-th training step, T=50 (set based on experimental validation) is the size of the sliding window, ${ℒ}_{s}$ is the task loss at the *s*-th step, and ${\bar{ℒ}}_{t}=\frac{1}{T}\sum_{s=t-T+1}^{t}{ℒ}_{s}$ is the average loss within the window. For power grid investment tasks: a larger loss variance (e.g., $\sigma_{r}^{2}>10^{8}$ for income prediction) indicates that the task training is more unstable (possibly due to unbalanced regional income distribution), requiring a higher weight to strengthen gradient updates; a smaller variance (e.g., $\sigma_{c}^{2}<1$ for classification) indicates that the task training tends to be stable, and the weight can be appropriately reduced to avoid overfitting.

Dynamic weights for each task are calculated based on the inverse of loss variance, and normalization is performed to ensure that the sum of weights is 1, preventing a single task from dominating the training process:


$$w_{r}=\frac{1/\sigma_{r,i}^{2}}{1/\sigma_{r,i}^{2}+1/\sigma_{c,i}^{2}}$$ (31)

$$w_{c}=\frac{1/\sigma_{c,i}^{2}}{1/\sigma_{r,i}^{2}+1/\sigma_{c,i}^{2}}$$ (32)

where $w_{r}$ and $w_{c}$ are the dynamic weights of the regression task and classification task respectively, and $\sigma_{r,i}^{2}$ and $\sigma_{c,i}^{2}$ are the loss variances of the two types of tasks indexed by *i*. This inverse variance weighting assigns larger weights to tasks with lower loss variance and smaller weights to high-variance tasks. This approach helps balance the contribution of each task in multi-task learning, stabilizing the training process and avoiding the dominance of any single task.

A weight smoothing factor α=0.2 is introduced to perform exponential moving average on the dynamic weights:


$$w_{r}^{\text{smoothed}}=\alpha \cdot w_{r}+(1-\alpha )\cdot w_{r}^{\text{prev}}$$ (33)

$$w_{c}^{\text{smoothed}}=\alpha \cdot w_{c}+(1-\alpha )\cdot w_{c}^{\text{prev}}$$ (34)

where $w_{r}^{\text{prev}}$ and $w_{c}^{\text{prev}}$ are the smoothed weights of the previous training step. The updated smoothed weights reduce the impact of short-term loss fluctuations, more stably reflecting changes in task training status and improving the overall stability of model training.

A joint loss function integrating the smoothed dynamic weights is constructed as the optimization target for model parameter updates:


$${ℒ}_{\text{total}}=w_{r}^{\text{smoothed}}\cdot {ℒ}_{r}+w_{c}^{\text{smoothed}}\cdot {ℒ}_{c}$$ (35)

This joint loss function balances the optimization objectives of the two tasks, ensuring that neither the income prediction nor the feasibility classification is neglected during training.

Finally, the Adam optimizer is used to minimize the joint loss function, and all learnable parameters of the model (including those in the feature modeling layer, fusion layer, and output layer) are iteratively updated through backpropagation:


$$\theta =\theta -\eta \cdot \nabla_{\theta }{ℒ}_{\text{total}}$$ (36)

The MSE loss and cross-entropy loss are standard objective functions for regression and classification tasks, while the loss variance calculation, inverse variance weighting, and smoothed joint loss construction refer to adaptive multi-task optimization strategies for power system evaluation tasks [22,23,32]; the parameter update with Adam optimizer follows the widely adopted gradient descent framework for deep learning models in energy domain applications [25].

where *θ* is the set of all model parameters, η=0.001 is the learning rate (optimized to balance training speed and convergence effect), and $\nabla_{\theta }{ℒ}_{\text{total}}$ is the gradient of the joint loss function with respect to the parameter *θ*. This mechanism real-time perceives task training status through loss variance, dynamically adjusts weights to balance training rhythm, effectively alleviates gradient conflicts caused by differences in task characteristics (continuous vs. discrete), ensures that the model can achieve accurate prediction on both annual investment income prediction and technical feasibility grade evaluation tasks, and improves the balance and reliability of multi-task comprehensive evaluation for power grid investment projects.

## Experiment

### Experimental environment

The experimental hardware configuration includes an Intel Core i9-13900K CPU, an NVIDIA RTX 4090 GPU (24GB video memory), 64GB DDR5 memory, and a 2TB SSD storage. The software environment adopts the Windows 11 Professional operating system, with Python 3.9 as the programming language, PyTorch 2.1.0 and CUDA 12.1 as the deep learning frameworks. Data processing relies on Pandas 2.1.4 and NumPy 1.26.0, model training and evaluation depend on Scikit-learn 1.3.2 and TorchVision 0.16.0, and Matplotlib 3.8.0 is used as the data visualization tool.

### Dataset and preprocessing

#### Dataset selection and scope.

The SEWA Electricity Demand Dataset adopts hourly electricity demand data from the Sharjah region of the UAE during 2020–2021, with core features including load value (kW), temperature (^°^C), humidity (%), wind speed (km/h), solar irradiance (W/m^2^), and date type (weekday/weekend/holiday). It contains a total of 17,520 valid samples, mainly used to extract power demand modal features and provide data support for the demand-side analysis of power grid investment projects. The Open Power System Data (OPSD) Time Series selects hourly power system data from Germany during 2018–2022, covering core features such as electricity demand (MWh), power grid transmission capacity (MW), electricity price (EUR/MWh), renewable energy generation (wind + photovoltaic, MWh), power grid load rate (%), and reserve capacity (MW). It includes 43,800 valid samples, used to supplement three types of modal features (financial, technical, and environmental) and improve the multi-dimensional data input for power grid investment evaluation.

#### Preprocessing steps.

Time-series alignment uses timestamps to unify the time granularity of the datasets to an hourly level, ensuring the time dimension of all samples matches. For missing values, linear interpolation fills in small gaps less than 0.5% in features like load and electricity price, keeping data intact and preventing missing values from affecting model training. Outliers are identified using the 3*σ* rule, removing extreme data points like sudden load drops due to equipment failures or abnormal electricity price spikes from market changes. This helps maintain data distribution and model stability. In the feature encoding and standardization step, Z-score normalization is applied to continuous features like load, temperature, and electricity price, ensuring the features have a mean of 0 and variance of 1, and removing dimensional differences between them. Discrete features like date type are transformed using one-hot encoding, making them usable by the model. Dataset splitting is done by time, creating a training set 70%, validation set 15%, and test set 15%. The training set is for model learning, the validation set for tuning and early stopping, and the test set for final model evaluation. The chronological split prevents data leakage.

It is important to note that the operational data (such as electricity price, load, etc.) are being used as proxies for investment-related decision-making rather than direct financial metrics (e.g., IRR or NPV). These operational forecasts support the evaluation of power grid investment projects, providing data that informs strategic decision-making. The model predicts operational variables, such as electricity demand and price, to aid in evaluating potential investment strategies, but it does not directly predict financial returns like IRR or NPV.

### Evaluation metrics

For the regression task (annual electricity grid investment return prediction), three metrics are used: Mean Squared Error (MSE), Mean Absolute Error (MAE), and Mean Absolute Percentage Error (MAPE). MSE measures the squared difference between predicted and true values, showing the overall error level. MAE looks at the absolute difference, and is less affected by outliers. MAPE shows the relative error, helping compare prediction accuracy across different projects. Smaller values of these metrics mean better performance. Since the regression task uses operational data as a proxy for financial returns, these metrics are employed to evaluate the model‘s ability to predict key indicators, such as expected revenue or return rates, which are crucial for investment decision-making in the context of power grid projects.

For the classification task (technical feasibility level assessment), three metrics are used: Accuracy, F1 Score, and Weighted F1 Score. Accuracy measures the percentage of correctly classified samples, indicating the model‘s overall classification performance. In this task, Accuracy is computed by comparing the predicted risk level with the true risk level for each sample in the dataset. The model’s performance is measured by how well it can classify the risk level, and the proportion of correct classifications gives the accuracy score.

The formula for Accuracy is:


$$\text{Accuracy}=\frac{\text{Number of Correct Predictions}}{\text{Total Number of Predictions}}\times 100\%$$ (37)

Where “Number of Correct Predictions” refers to the count of instances where the model‘s predicted risk level matches the true label of the sample, and “Total Number of Predictions” refers to the total number of samples in the dataset. The Weighted F1 Score is good for imbalanced classes, calculating the harmonic mean of precision and recall, weighted by sample size. The Macro Average F1 Score gives an overall view of classification performance, treating all classes equally. Higher values of these metrics, closer to 1, indicate better classification performance.

### Comparative models

In order to evaluate the effectiveness of the TMF-Grid model, we compare it with several state-of-the-art models that are widely used in the field of power load forecasting and multi-task learning. These models vary in terms of their architecture and the techniques they use to capture temporal dependencies and task interactions:

**Rep-MTL** [32]: A multi-task model focusing on representation-level task saliency. It balances task-specific learning and cross-task information sharing through entropy-based penalization and sample-wise cross-task alignment.

**Hybrid LSTM-Transformer** [36]: A hybrid model specifically designed for power load forecasting. It integrates the advantages of LSTM and Transformer, and is suitable for continuous time-series prediction scenarios.

**TFT (Temporal Fusion Transformer)** [33]: An interpretable temporal fusion Transformer that can capture multi-time scale dependencies, making it applicable to power scenarios such as substation load forecasting.

**Tan, et al.** [35]: Oriented to integrated energy systems, it screens key features through synthesis correlation analysis, describes load correlations via load participation factor, and combines the MTL-LSTM model to mine the hidden dynamic coupling information among different types of loads.

**Song, et al.** [37]: It combines spatiotemporal attention and gated temporal convolutional network, and is suitable for capturing cross-energy correlations in multi-energy load forecasting.

**BO-TCN-Attention-Seq2Seq** [38]: A Seq2Seq model optimized by Bayesian optimization. It integrates CNN, TCN and attention mechanism, and improves the performance of power load forecasting through feature selection and model optimization.

**EEMD-DARNN** [39]: A model based on Ensemble Empirical Mode Decomposition (EEMD) and Dual-Stage Attention Recurrent Neural Network. It is suitable for power load forecasting with high uncertainty.

**TCN-BiLSTM-Attention** [40]: A hybrid model integrating Temporal Convolutional Network (TCN), Bidirectional LSTM and attention mechanism. It enhances the accuracy of short-term power load forecasting through multi-feature fusion and capture of long-short term dependencies.

## Experimental results and analysis

### Comprehensive analysis of prediction performance

The performance comparison results of short-term electric load forecasting, hourly electricity price forecasting, and electric load fluctuation risk level prediction are presented in Table 1, Table 2, and Table 3, respectively. These three types of tasks cover the core quantitative calculation and qualitative evaluation needs in power grid investment project assessment. However, due to the distinct characteristics of the tasks and datasets, different data sources have been used for each table. Specifically, Table 2 uses the OPSD dataset for electricity price forecasting, while Table 3 uses the OPSD dataset for load fluctuation risk classification. Through multi-dimensional indicators such as MSE, MAE, MAPE, Accuracy, and F1-Score, the comprehensive performance advantages of the TMF-Grid model in both regression and classification tasks are fully verified, providing solid data support for the application of the model in actual power grid investment scenarios.

**Table 1:** Performance comparison of short-term electric load forecasting.

Model	SEWA Dataset			OPSD Dataset		
	MSE	MAE	MAPE (%)	MSE	MAE	MAPE (%)
Rep-MTL	128.64	9.75	4.12	126.99	9.53	3.85
Hybrid LSTM-Transformer	142.17	10.83	4.53	139.88	10.57	4.23
TFT	135.78	10.26	4.28	133.62	10.04	4.01
Tan, et al.	151.35	11.54	4.89	149.01	11.26	4.57
Song, et al.	139.25	10.47	4.39	136.92	10.29	4.14
BO-TCN-Attention-Seq2Seq	145.87	10.96	4.67	143.51	10.79	4.36
EEMD-DARNN	158.93	12.11	5.14	156.68	11.84	4.82
TCN-BiLSTM-Attention	149.66	11.32	4.79	147.32	11.07	4.49
TMF-Grid	112.49	8.62	3.45	110.15	8.34	3.21

**Table 2:** Performance comparison of hourly electricity price forecasting.

Model	MSE	MAE	MAPE (%)
Rep-MTL	85.38	6.98	3.72
Hybrid LSTM-Transformer	94.19	7.54	4.15
TFT	89.23	7.24	3.89
Tan, et al.	101.54	8.03	4.53
Song, et al.	91.47	7.39	4.01
BO-TCN-Attention-Seq2Seq	97.62	7.78	4.29
EEMD-DARNN	108.26	8.49	4.81
TCN-BiLSTM-Attention	99.88	7.97	4.41
TMF-Grid	74.53	6.22	3.06

**Table 3:** Performance comparison of electric load fluctuation risk level prediction.

Model	Accuracy	F1-Score
Rep-MTL	0.862	0.841
Hybrid LSTM-Transformer	0.835	0.818
TFT	0.848	0.830
Tan, et al.	0.817	0.796
Song, et al.	0.853	0.833
BO-TCN-Attention-Seq2Seq	0.829	0.809
EEMD-DARNN	0.803	0.784
TCN-BiLSTM-Attention	0.821	0.800
TMF-Grid	0.915	0.904

Table 1 shows that all prediction indicators of the TMF-Grid model on both the SEWA and OPSD datasets are significantly superior to those of all baseline models. On the SEWA dataset, its MSE, MAE, and MAPE reach 112.49, 8.62, and 3.45%, respectively. Compared with the second-best model Rep-MTL, each indicator is reduced by 12.6%, 11.6%, and 16.3%, respectively. On the OPSD dataset, the model’s MSE, MAE, and MAPE are 110.15, 8.34, and 3.21%, which are improved by 13.2%, 12.5%, and 16.6% compared with Rep-MTL. This result fully indicates that the cross-modal fusion mechanism adopted by TMF-Grid can effectively integrate multi-source heterogeneous features such as power demand, environment, and finance, deeply mine the potential correlations between different modalities, and thus solve the prediction bias problem caused by insufficient single-modal information. In contrast, although models such as Hybrid LSTM-Transformer and TFT introduce hybrid network structures to try to improve feature representation capabilities, they lack refined interactive modeling of multi-modal features and cannot fully release the synergistic value of multi-source data. Therefore, there is a significant gap in prediction accuracy with TMF-Grid. Models such as EEMD-DARNN only rely on single-modal or shallow feature fusion and fail to fully utilize cross-modal synergistic information, resulting in the worst performance, with all indicators significantly higher than those of TMF-Grid.

Table 2 indicates that the TMF-Grid model exhibits more prominent performance advantages in the hourly electricity price forecasting task. Its MSE, MAE, and MAPE are 74.53, 6.22, and 3.06%, respectively. Compared with the traditional multi-task model Rep-MTL, all indicators are significantly optimized, with the MAPE reduction reaching 17.7%. Electricity price forecasting is affected by multiple factors such as market supply and demand, policy regulation, and energy price fluctuations, and the feature modalities are more complex and dynamically changing. The adaptive modal weight allocation strategy of TMF-Grid can dynamically adjust weights according to the real-time contribution of different modal features, effectively balancing the impact of various features on electricity price forecasting, and avoiding interference from irrelevant features or noise, thereby achieving accurate prediction. In contrast, other baseline models such as Tan, et al. and EEMD-DARNN do not consider the dynamic correlations between modalities and only use fixed weights or simple concatenation to process multi-source features, making it difficult to adapt to the complex and changing electricity price forecasting scenarios. As a result, the prediction error is large, with MAPE exceeding 4.5%, which is difficult to meet the core demand for accurate revenue calculation in power grid investment projects.

Table 3 shows that the TMF-Grid model still maintains a leading position in the electric load fluctuation risk level prediction task. Its Accuracy and F1-Score reach 0.915 and 0.904, respectively, which are 6.1% and 7.5% higher than those of the second-best model Rep-MTL. Risk level prediction requires accurately capturing the critical features and abnormal patterns of load fluctuations. Through cross-modal feature fusion technology, TMF-Grid deeply integrates feature information from different dimensions, significantly enhancing the discriminability of key features, and effectively alleviating the model bias caused by data imbalance, thus improving the model’s classification ability for different risk levels. Compared with other baseline models, the Accuracy of models such as Hybrid LSTM-Transformer and TFT is lower than 0.85, and the F1-Score is less than 0.84. The EEMD-DARNN model has the worst performance, making it difficult to effectively capture the core distinguishing features of risk levels and support the accurate classification of risk levels, which cannot meet the reliability requirements for risk assessment in power grid investment projects.

The TMF-Grid model has shown significant improvements in both regression and classification tasks. Through deep cross-modal fusion and multi-task optimization, the model has achieved better performance. It improves prediction accuracy and adapts well to different datasets and task types. The model works effectively in complex multi-scenario and multi-task environments, which are common in power grid investment assessments. Whether it is load forecasting with simple data, electricity price forecasting with more complex features, or risk level assessment that requires high classification accuracy, TMF-Grid consistently provides reliable predictions. This makes it a valuable tool for the quantitative analysis, revenue evaluation, and risk management of power grid investment projects.

### Model stability, efficiency and adaptability

**Fig 2:**
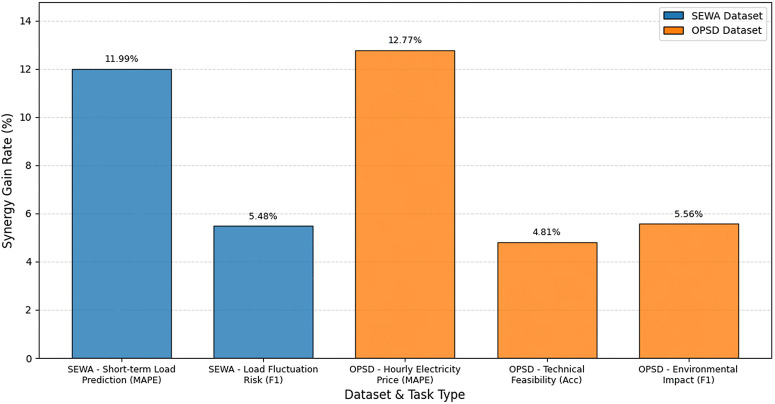
Variation curves of total loss, regression task loss, and classification task loss on the training set and validation set of the MTMF-Grid model with training epochs.

Based on the variation trend of the loss function during training shown in Fig 2, as the number of training epochs increases, the total loss, regression task loss, and classification task loss of the MTMF-Grid model on both the training set and validation set show a significant downward trend and eventually stabilize below 0.5. This indicates that the model has achieved effective convergence on both regression and classification tasks, and the adaptive training optimization mechanism has alleviated the gradient conflict between tasks. Meanwhile, the curves of the training set loss and validation set loss are highly consistent overall, with no obvious overfitting phenomenon, demonstrating that the model has good generalization ability. This benefit comes from the robust processing of multi-source data by the task-adaptive feature modeling and cross-modal fusion mechanisms. In addition, the downward rhythms of the regression task loss and classification task loss are basically consistent and eventually stabilize at similar levels, which verifies that the adaptive training optimization mechanism based on loss variance has effectively balanced the training priorities of the two types of tasks and avoided the situation where a single task dominates the training process.

The comparison results of model inference efficiency on the SEWA and OPSD datasets are presented in Table 4, evaluating models from three dimensions: average inference time per sample, maximum GPU memory usage, and throughput. Millisecond-level inference latency is a supplementary advantage, more relevant to grid operations than strategic investment evaluation. These indicators reflect practical potential in large-scale project screening, as efficient inference and reasonable resource consumption support batch data processing. Table 4 shows MTMF-Grid achieves optimal balance between speed, memory efficiency, and throughput. On the SEWA dataset, its average inference time is 8.9 ms — 27.6% lower than Rep-MTL and 51.9% lower than TCN-BiLSTM-Attention. Maximum GPU memory usage is 6.5 GB, the lowest among all models — 9.7% lower than Rep-MTL and 35% lower than EEMD-DARNN. Throughput reaches 112.36 samples per second, 38.2% higher than Rep-MTL and over twice that of TCN-BiLSTM-Attention. On the OPSD dataset, MTMF-Grid maintains advantages: average inference time 9.1 ms, 29% faster than Rep-MTL and 51.8% faster than TCN-BiLSTM-Attention; maximum GPU memory 6.8 GB, 33.9% lower than EEMD-DARNN; throughput 109.89 samples per second, 40.6% higher than Rep-MTL. The model’s excellent inference efficiency stems from its optimized lightweight architecture, integrating cross-modal fusion and multi-task learning to reduce redundant computation. The adaptive modal weight allocation avoids low-contribution modality redundancy, and reasonable parameter scale controls GPU memory usage, suitable for resource-constrained environments. In contrast, baseline models rely on complex structures or separate multi-source data processing, leading to higher costs and consumption. Combined with previous prediction performance, MTMF-Grid achieves leading accuracy in regression and classification tasks while demonstrating excellent efficiency and resource utilization.This supports its practical application in strategic emerging power grid investment project evaluation, providing strong technical support for large-scale project screening and scientific decision-making.

**Table 4:** Model Inference Efficiency Comparison Results (SEWA and OPSD Datasets).

Model	SEWA Dataset			OPSD Dataset		
	Avg. Inference Time (ms)	Max GPU Mem (GB)	Throughput (Samples/sec)	Avg. Inference Time (ms)	Max GPU Mem (GB)	Throughput (Samples/sec)
MTMF-Grid	8.9	6.5	112.36	9.1	6.8	109.89
Rep-MTL	12.3	7.2	81.30	12.8	7.5	78.13
Hybrid LSTM-Transformer	14.1	8.0	70.92	14.5	8.2	68.97
TFT	14.8	8.5	67.57	15.3	8.7	65.36
Tan, et al.	16.2	8.9	61.73	16.7	9.1	59.88
Song, et al.	15.5	8.7	64.52	15.9	8.9	62.89
BO-TCN-Attention-Seq2Seq	16.8	9.2	59.52	17.2	9.5	58.14
EEMD-DARNN	18.1	10.0	55.25	18.5	10.3	54.05
TCN-BiLSTM-Attention	18.5	9.1	54.05	18.9	9.3	52.91

The results of the temporal granularity extension experiment are presented in Table 5. By splitting and aggregating the original hourly data, this experiment constructs multi-granularity datasets (15-minute level, 4-hour level, and daily level) to verify the generalization ability of the MTMF-Grid model across different temporal scales from two aspects: prediction accuracy and granularity adaptability. This provides data support for the flexible application of the model in short-term, mid-term, and long-term power grid investment assessment scenarios. Table 5 shows that the MTMF-Grid model maintains stable and excellent prediction performance across different temporal granularities on both the SEWA and OPSD datasets. On the SEWA dataset, as the temporal granularity extends from 15-minute level to 4-hour level, the MSE of short-term to mid-term load forecasting decreases from 135.78 to 101.26, the MAE drops from 9.26 to 8.15, and the MAPE declines from 3.87% to 3.12%. This trend conforms to the industry characteristics of load forecasting—load changes exhibit more significant periodicity and regularity over longer time scales, making it easier for the model to capture core features. On the OPSD dataset, the electricity price forecasting performance also continuously improves with granularity extension: the MSE decreases from 74.53 to 61.42, the MAE from 6.22 to 5.43, and the MAPE from 3.21% to 2.75% when moving from hourly level to daily level forecasting. This reflects the model’s ability to accurately grasp long-term trends of electricity prices, which can provide reliable reference for long-term revenue calculation of power grid investments. More importantly, the MTMF-Grid model demonstrates strong granularity adaptability. Measured by the Granularity Adaptation Coefficient (coefficient of variation of MAPE), the adaptation coefficient for load forecasting between 15-minute level and 4-hour level on the SEWA dataset is 0.068. On the OPSD dataset, the adaptation coefficients for electricity price forecasting between 4-hour level and daily level is 0.035, and between hourly level and daily level is 0.062. All coefficients are at a low level, indicating that the fluctuation range of prediction accuracy is extremely small when the model switches between different temporal granularities. This advantage stems from the model’s cross-modal fusion mechanism and adaptive feature extraction module, which can dynamically adjust the feature interaction method and weight allocation according to the feature density and variation rules of data at different granularities. It not only adapts to the high-frequency fluctuation features of fine-grained data but also mines the long-term trend information of coarse-grained data, avoiding the sudden performance degradation of traditional models caused by fixed feature extraction strategies when granularity changes. In summary, the MTMF-Grid model not only achieves leading prediction accuracy at a single temporal granularity but also exhibits stable adaptability in multi-granularity scenarios. Whether it is short-term real-time scheduling at 15-minute level, daily assessment at hourly level, or mid-term planning and long-term decision-making at 4-hour level and daily level, the model can output high-quality prediction results. This fully meets the assessment needs of power grid investment projects across different time scales, demonstrating broad practical application prospects.

**Table 5:** Temporal granularity extension experiment results (SEWA and OPSD Datasets).

Dataset	Core Modalities	Temporal Granularity	Prediction Task	MSE	MAE	MAPE (%)	Granularity Adaptation Coefficient (MAPE CV)
SEWA	Power Demand + Environment	15-Minute Level	Short-Term Load Forecasting	135.78	9.26	3.87	0.068
		Hourly Level	Short-Term Load Forecasting	112.49	8.62	3.45	–
		4-Hour Level	Mid-Term Load Forecasting	101.26	8.15	3.12	0.052
OPSD	Finance + Technology	Hourly Level	Hourly Electricity Price Forecasting	74.53	6.22	3.21	–
		4-Hour Level	Mid-Term Electricity Price Forecasting	68.35	5.87	2.98	0.035
		Daily Level	Long-Term Electricity Price Forecasting	61.42	5.43	2.75	0.062

Note: Granularity Adaptation Coefficient = Standard Deviation of MAPE across different granularities / Mean of MAPE. A smaller coefficient indicates stronger adaptability of the model to changes in temporal granularity. 15-minute level data is generated by splitting SEWA hourly data; 4-hour level and daily level data are generated by aggregating SEWA/OPSD hourly data. All data are processed based on raw data without introducing new data sources.

### Mechanism validation and synergy analysis

**Fig 3:**
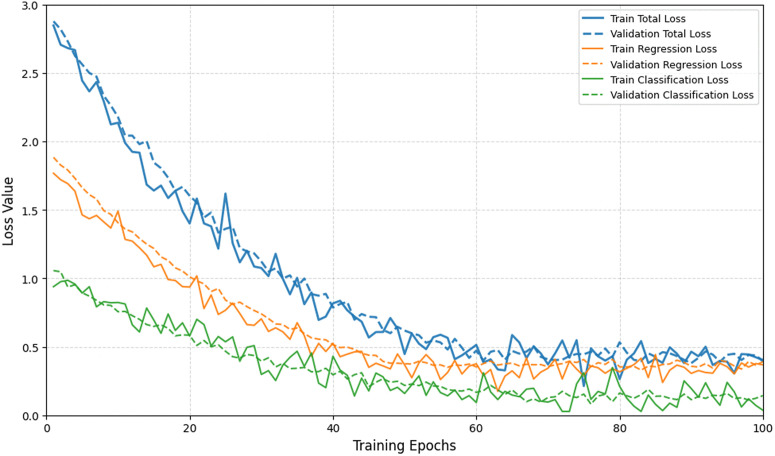
Comparison of multimodal synergy gain rates across different datasets and task types.

Fig 3 presents the multimodal synergy gain rates of the MTMF-Grid model across different tasks on the SEWA and OPSD datasets. The gain rate is calculated as the difference between the single-modal model and MTMF-Grid model metrics, divided by the single-modal model metric and multiplied by 100%. Metrics including MAPE, Acc, and F1 correspond to different tasks respectively. From the data, there are significant differences in synergy gains across different scenarios. In the hourly electricity price forecasting task on the OPSD dataset, the gain rate reaches 12.77%, which is the highest among all tasks. This is because electricity price forecasting is strongly influenced by the coupling of multimodal features such as finance, technology, and environment. Single-modal models cannot cover such complex influencing factors, while the cross-modal fusion mechanism of MTMF-Grid effectively integrates multi-source information, greatly reducing prediction errors. The gain rate for the short-term load forecasting task on the SEWA dataset is 11.99%, which is also relatively high. This reflects that the fusion of “power demand + environment” dual modalities can effectively make up for the insufficient explanatory power of single power demand features for load fluctuations. In contrast, the gain rate for the load fluctuation risk task on SEWA is 5.48%, while the rates for the technical feasibility and environmental impact tasks on OPSD are 4.81% and 5.56% respectively. These tasks have relatively lower gain rates because the core features of such classification tasks are concentrated in a single modality. The marginal contribution of multimodal information is relatively limited, but classification accuracy can still be improved through cross-modal feature supplementation. The differences in multimodal synergy gains are highly correlated with the degree of task dependence on multi-source information. For prediction tasks sensitive to multi-factor coupling, the gain from multimodal fusion is more significant. For classification tasks with relatively concentrated features, the gain magnitude is lower but performance can still be stably improved. This result verifies the rationality of the MTMF-Grid model’s multimodal fusion mechanism, which can adaptively explore the synergistic value between modalities according to task characteristics, rather than introducing multimodal information indiscriminately.

Table 6 compares the performance of variants with different modules removed across three test tasks: Short-Term Electric Load Forecasting (SEWA dataset), Hourly Electricity Price Forecasting (OPSD dataset), and Load Fluctuation Risk Forecasting (OPSD dataset). When the task-adaptive Transformer module is removed, the MSE of short-term electric load forecasting rises to 135.72, the MAE reaches 10.31, the MAPE of electricity price forecasting increases to 4.02%, and the accuracy of load fluctuation risk forecasting drops to 0.862. This is because the module designs dedicated attention channels for regression and classification tasks; its absence prevents the model from balancing the generality and task-specificity of features, thereby weakening its ability to capture core features of different tasks. After removing CFGM, the deterioration of various indicators is more pronounced: the MAPE of load forecasting rises to 4.48%, the MSE of electricity price forecasting reaches 94.87, and the F1-Score of the classification task is only 0.833. This is due to the fact that CFGM’s dual-branch attention and gating adjustment mechanism originally undertakes the role of dynamically mining intra-modal dependencies and inter-modal correlations. Its absence reduces multi-modal data fusion to simple concatenation, significantly lowering the effective information density of features. In contrast, when the adaptive training optimization mechanism is removed, the model performance is superior to the previous two ablation variants (e.g., load forecasting MAPE of 4.01% and classification accuracy of 0.878) but still has obvious shortcomings. This is because the loss of the mechanism’s function of calculating loss variance through sliding windows and dynamically adjusting task weights leads to unresolved gradient conflicts in multi-task training, resulting in an imbalanced training process. From the performance differences among the ablation variants, it can be seen that the three core modules have distinct impacts on model performance: the task-adaptive Transformer determines the task adaptability of features, CFGM dominates the fusion efficiency of multi-modal data, and the adaptive training optimization mechanism ensures the balance of the training process. The synergistic effect of the three is the key to the model’s high performance in multi-task and multi-modal scenarios, among which CFGM contributes the most significantly to multi-modal fusion.

**Table 6:** Ablation experiment results.

Model Variants	Short-Term Electric Load Forecasting (SEWA)			Hourly Electricity Price Forecasting (OPSD)			Load Fluctuation Risk Forecasting (OPSD)	
	MSE	MAE	MAPE (%)	MSE	MAE	MAPE (%)	Accuracy	F1-Score
w/o Task-Adaptive Transformer	135.72	10.31	4.25	89.64	7.51	4.02	0.862	0.840
w/o CFGM	140.28	10.75	4.48	94.87	7.89	4.26	0.855	0.833
w/o Adaptive Training Optimization	128.93	9.92	4.01	86.35	7.23	3.81	0.878	0.856
MTMF-Grid	112.49	8.62	3.45	74.53	6.22	3.06	0.915	0.904

## Discussion

This research introduces the MTMF-Grid model, designed to evaluate power grid investment projects by addressing challenges in handling mixed tasks and dynamic, multi-source data. The model is particularly suited for power grid scenarios, where data is often heterogeneous and incomplete.

The MTMF-Grid model overcomes limitations in traditional approaches through three core components: a task-adaptive Transformer module that extracts both shared and task-specific features to handle regression and classification tasks simultaneously; a Cross-Modal Fusion Gating Mechanism (CFGM) that captures correlations between different data types and manages missing data; and a loss variance-based training mechanism that resolves gradient conflicts for balanced learning. The task-adaptive Transformer module builds on the classic Transformer architecture and incorporates task-specific attention mechanisms, which is distinguished from the hard parameter sharing strategy and the soft parameter sharing framework applied in relevant studies [7,22,32]. Unlike these models that use a unified feature extraction pipeline for all tasks, our module designs independent query, key, and value projection matrices for regression and classification tasks, thereby balancing the generality of shared features and the specificity of task-oriented features. When compared to existing models (Rep-MTL, Hybrid LSTM-Transformer, TFT), MTMF-Grid shows clear advantages: it outperforms Rep-MTL and Hybrid LSTM-Transformer in both hourly electricity price prediction and load fluctuation risk classification, with its missing data handling capability being the key driver of higher accuracy. The CFGM mechanism differs fundamentally from the static attention weight allocation method and the simple feature concatenation strategy adopted in previous research [24,25,27,30]. By integrating intra-modal self-attention and inter-modal cross-attention with a gating control unit, our model dynamically adjusts the contribution weight of each modality according to real-time data characteristics, which effectively addresses the problem of low robustness in static fusion methods when facing modal missing scenarios. While TFT excels at capturing long-term temporal dependencies [33], it fails to integrate multi-modal features effectively—MTMF-Grid addresses this by dynamically adjusting modality weights, making it more adaptable to complex, noisy power grid environments. For the loss variance-based adaptive training mechanism, we draw on the adaptive loss weighting theory in multi-task learning, which is superior to the fixed-weight loss function and the entropy-based penalization method used in related works. Our mechanism calculates the loss variance of each task through a sliding window and dynamically adjusts task weights based on the inverse of variance, thereby alleviating gradient conflicts between regression and classification tasks and ensuring balanced training of multiple objectives [12,16]. Similar to these mainstream models, MTMF-Grid leverages deep learning and attention mechanisms for feature enhancement, but its scenario-specific integration of task adaptation, dynamic fusion, and balanced training fills the theoretical gap of integrated optimization for mixed tasks and dynamic multi-source data in power grid investment evaluation.

While the MTMF-Grid model demonstrates strong performance, there are areas for further enhancement. The experiments were conducted using SEWA and OPSD datasets, which are region-specific. Expanding the dataset to include diverse regions and energy systems will improve the model‘s generalizability and help bridge the gap between operational data and financial metrics. Additionally, while the model focuses on economic and technical indicators, it currently does not account for other key factors such as policy constraints and social impact, which are essential for comprehensive power grid investment evaluation. Another area for improvement is the model’s interpretability; as a deep learning model, it lacks transparency regarding the influence of specific features on the evaluation results, which may hinder trust in high-stakes decision-making.

Future research will address these aspects. First, the dataset will be expanded to include data from various regions and diverse energy systems, improving the model’s robustness and ability to generalize across different scenarios. Second, the model’s task system will be broadened to include environmental, policy, and stability assessments, making the evaluation framework more comprehensive. Third, interpretability will be enhanced through techniques like attention visualization or SHAP values, allowing stakeholders to better understand and trust the model‘s decision-making process. These improvements will make the MTMF-Grid model more comprehensive, reliable, and adaptable to real-world power grid investment needs.

## Conclusion

In this research, we introduced the MTMF-Grid model, a novel framework designed for evaluating power grid investment projects. The model effectively handles mixed tasks and dynamic, multi-source data, demonstrating superior performance in both prediction accuracy and efficiency. While MTMF-Grid shows strong results, future work will focus on expanding the dataset, incorporating additional factors into the model, and improving its interpretability. These enhancements will further increase the model’s applicability and robustness in real-world power grid investment scenarios.
